# Clinical internship experience of African undergraduate nursing students in China: a descriptive qualitative study

**DOI:** 10.1186/s12909-025-06860-1

**Published:** 2025-02-15

**Authors:** Lingxi Chen, Chao Xu, Yunxian Zhou

**Affiliations:** School of Nursing, Zhejiang Chinese Medical University, 548 Binwen Road, Binjiang District, Hangzhou, Zhejiang 310053 China

**Keywords:** Clinical internship, African undergraduate students, Nursing, China, Qualitative, Interviews

## Abstract

**Background:**

Clinical internships are crucial in nursing education; however, they often present challenges that can elicit negative emotions among students, especially among international nursing undergraduates. Despite its importance, there is a lack of research on the experiences of African nursing undergraduates during clinical internships in China.

**Aim:**

To explore the clinical internship experiences of African undergraduate nursing students within the Chinese healthcare system.

**Method:**

A descriptive qualitative study design was adopted. Thirteen African undergraduate nursing students with over three months of clinical internship experience in China were recruited through purposive sampling. Semi-structured interviews were conducted to collect data on their experiences. Conventional content analysis was used for data analysis.

**Results:**

Five categories and twelve subcategories were identified through the analysis: multiple challenges (language is an issue, identity embarrassment, peer pressure); accepting challenges (having to accept, being able to accept); making personal efforts (self-image managing, self-reflecting and improving); being not alone (preceptors are strong backings, mutual assistance among fellow students, patients provide trust and warmth); and growing up as a real nurse (increasing nursing knowledge and skills, enhancing nursing professional quality).

**Conclusions:**

The findings offer nursing colleges and hospitals a deeper understanding of the specific challenges encountered by African undergraduate nursing students during their clinical internships in China. This study also highlights the students’ handling of complex issues and the subsequent positive changes they experienced.

## Introduction

With the enhancement of China’s comprehensive national strength and the promotion of the “belt and road initiative”, the international education industry in China is flourishing. China has emerged as the most favored education destination in Asia for international students and ranks third globally [1, 2]. In 2018, a total of 492, 200 international students from 196 countries and regions were studying in China [2], including a significant number of international undergraduate nursing students. Unlike high-income countries with diverse international student populations, China primarily attracts undergraduate nursing students from developing nations, with African students forming a substantial group. These students pursue nursing studies in China due to factors such as lower tuition fees or the desire for overseas study in a country with advanced healthcare technology [3].

Clinical internships are a fundamental component of undergraduate nursing education, enabling students to apply theoretical knowledge into practice and to develop their clinical thinking abilities and competencies in patient care [4, 5]. African nursing undergraduates in China complete nursing and Chinese language courses during their first three years, followed by a 10-month internship at the university’s affiliated hospitals and community health centers.

Clinical practice is recognized as one of the most challenging aspects of learning for international nursing students [6], including those from Africa studying in China [7]. Previous studies have indicated that these students experience significant stress during their clinical internships [8, 9], significantly affecting their physical and mental well-being, as well as their overall satisfaction with their study abroad experience. Common challenges include language barriers, cultural differences, a lack of practical experience, and unfamiliarity with the healthcare system [10–12]. These negative experiences can even lead students to reconsider their career choice [13].

Gaining insights into the clinical internship experiences of African undergraduate nursing students is crucial for staff to develop targeted interventions that enhance student satisfaction. To date, most related studies have focused on Western developed countries, while the clinical internship experience of African undergraduate nursing students in China remain under-explored. China, with its emphasis on “group orientation” and collectivist culture, presents a unique context [14]. Given the cultural and educational differences, findings from previous studies may not be directly applicable to African nursing undergraduates in China. Thus, this study aims to use descriptive qualitative research to explore the internship experiences from the perspective of African nursing students in China.

## Method

### Design

A descriptive qualitative research design was adopted in this study. This approach tends to draw from the general tenets of naturalistic inquiry, allowing the target phenomenon to be studied in its natural context, as if the researchers were not present [15].

### Participants

Purposive sampling was used to recruit participants who could provide rich information and met the inclusion criteria from a medical university in Zhejiang, China. The university has three affiliated tertiary hospitals. The inclusion criteria were: (1) African nursing students studying in China; (2) proficiency in English communication; and (3) having completed more than 3 months of clinical internship experience. Students who were unwilling to participate in an interview were excluded.

Two researchers (YZ and CX) identified eligible students. Subsequently, CX contacted potential participants via text message to explain the study and invite them to an in-person interview. Of the sixteen African nursing students invited, three declined due to scheduling conflicts.

### Data collection

A preliminary interview guide was constructed based on a review of the literature related to the clinical internship experiences of African nursing students by the researcher (LC). LC is a female PhD candidate in nursing who has previously published several qualitative papers in English. This guide was subsequently reviewed and revised by qualitative expert YZ, a female nursing professor with a PhD from Australia. The revised interview guide is shown in Table 1.

**Table 1:** Interview guide

Interview guide
1. How is your clinical practice going so far?
2. Please describe some impressive experiences or stories while you were having clinical practice
3. Could you tell me any challenges you met when you were having your clinical practice?
4. How do you cope with the challenges you encountered?

Data collection was conducted in English by researcher CX, a male postgraduate nursing student with comprehensive training in qualitative studies and strong English communication skills. Three face-to-face interviews were conducted in quiet, private classrooms or meeting rooms. Due to the COVID-19 pandemic, two telephone interviews and eight video interviews were also conducted. During each interview, only the participant and the researcher were present. The interviews were audio recorded, with an average length of 45 min. Field notes were promptly written after each interview to record nonverbal observations (e.g. environment, body language).

### Data analysis

The audio recordings were transcribed verbatim into English within 24 h by researcher CX. The transcripts were then returned to the participants for their verification. Data analysis and collection were conducted simultaneously. A conventional content analysis [16] was used to analyze the interview transcripts by two coders (LC and CX). The whole process of data analyses was guided by YZ. Disagreements were resolved through discussion among the three researchers until a consensus was reached. English was the language utilized for analysis.

Firstly, the two coders (LC and CX) independently read the transcripts multiple times to immerse themselves in the data and gain a comprehensive understanding of the participants’ experience. Next, narrative data related to clinical internship experiences were hand-coded line by line by the coders (LC and CX) independently. Following this, the codes were categorized into broader categories and subcategories based on shared characteristics. Finally, definitions for each category and subcategory were developed, and supportive quotes from the transcripts were selected. Data saturation, which was defined as the point at which no new subcategories or categories emerged, was reached during the 11th interview. Two subsequent interviews were conducted to confirm that the data was repetitive and yielded no new insights.

### Rigour

To maintain rigour in this study, several strategies were adopted: (1) the researchers maintained an open-minded approach and practiced continuous reflexivity, by writing reflective journals to minimize the influence of pre-existing understandings or potential biases on the research findings, especially given the familiarity with participants; (2) A trusting relationship was established between the researcher (CX) and the participants through daily greetings and assurance of confidentiality. CX was previously acquainted with six participants and had interacted with the other seven via WeChat before the interviews; (3) Eight participants engaged in the member checking process, where analysis results were shared with them to ensure the consistency with their actual experiences.

### Ethical considerations

This study received approval from the Medical Ethical Committee of Zhejiang Chinese Medical University. Written or audio-recorded verbal consent was obtained from all participants before each interview. Participants were informed of their right to withdraw from the study at any time. To ensure confidentiality, all identifying information related to the participants (e.g. their names, addresses) was anonymized.

## Results

A total of 13 African undergraduate nursing students (9 females and 4 males), aged 20–29 years, studying in China participated in this study. Detailed demographic and general information of the participants are shown in Table 2.

**Table 2:** Key characteristics of study participants

Participant	Gender	Age	Mother language	Nationality	Internship duration (month)
P1	Male	25	English	Zambia	10
P2	Female	26	English	Zambia	10
P3	Female	24	English	Zambia	10
P4	Male	22	English	Ghana	6
P5	Male	27	English	Gabon	7
P6	Female	24	English	Zimbabwean	6
P7	Female	29	French	Gabon	5
P8	Female	24	French	Chad	6
P9	Male	23	English	Uganda	7
P10	Female	20	English	Nigeria	8
P11	Female	21	English	Gambia	8
P12	Female	21	English	Gambia	8
P13	Female	20	English	Zambia	9

The analysis identified five categories and 12 subcategories, encapsulating the overall clinical internship experiences of the participants, as detailed in Table 3. These categories were presented as follows: (1) “multiple challenges” (2) “accepting challenges” (3) “making personal efforts” (4) “being not alone” and (5) “growing up as a real nurse”.

**Table 3:** Categories and subcategories of the study

Categories	Subcategories
Multiple challenges	Language is an issue
	Identity embarrassment
	Peer pressure
Accepting challenges	Having to accept
	Being able to accept
Making personal efforts	Self-image managing
	Self-reflecting and improving
Being not alone	Preceptors are strong backings
	Mutual assistance among fellow students
	Patients provide trust and warmth
Growing up as a real nurse	Increasing nursing knowledge and skills
	Enhancing nursing professional quality

### Multiple challenges

“Multiple challenges” refers to the difficulties that participants encountered during their clinical internships. The internships within the Chinese healthcare system were more complex than the undergraduates had anticipated. Language barriers posed a significant obstacle. Furthermore, being labeled as “foreigners” and “interns”, coupled with peer pressure, created challenging situations and induced negative emotions.

#### Language is an issue

Most patients and their family members primarily communicated in Chinese, with some elderly patients using local dialects exclusively. However, the majority of participants were native English speakers with limited proficiency in Chinese. These language barriers caused stress, particularly in the early stages of their internships.

*When we started our first days, it was kind of stressful because we don’t know how to communicate with the patients. (P4)*

Clinical practice required a thorough understanding of Chinese medical terminology, which was unfamiliar to the participants. Consequently, they struggled with verifying medications before administration and independently collecting and recording medical histories, hindering the establishment of trusting relationships with patients.

*I don’t know Chinese. That’s why most patients will be a bit scared for you to do the nursing procedures on them because they think you don’t know the medicine you are administering. (P13)*

Due to the participants’ limited Mandarin proficiency, clinical preceptors attempted to teach in English. However, some participants noted that certain supervisors needed to improve their English skills, as inadequate language skills led to misunderstandings and inefficiencies.

*They try to teach you some practical skills, but they don’t really know how to express it in English. (P10)*

#### Identity embarrassment

Compared to Chinese individuals, the African nursing students have distinct physical characteristics, such as skin color and body shapes, which drew excessive attention and comments during their clinical internships. Although participants understood this attention was driven by curiosity rather than malice, incidents like unauthorized photography and being called “black people” made them feel uncomfortable and disrespected.

*They take pictures of us. I don’t like it. It’s uncomfortable. (P12)*

The participants’ international identity affected patient trust, as some Chinese patients held stereotypes about the professional competence of students from developing countries. Furthermore, as nursing interns without professional qualifications, the participants were often rejected by patients. These rejections and the associated distrust generated the feeling of self-doubt and even reluctance to perform nursing procedures.

*They don’t want you to do any procedures on them. It reduces your confidence. You feel like, am I not good or what’s wrong? (P5)*

#### Peer pressure

The high performance of some fellow international nursing students created stress for some participants. Chinese preceptors tended to publicly praise students who displayed strong Chinese proficiency and nursing skills. This behaviour made other students feel unfairly compared, and alienated.

*So some teachers, they used to compare… they would tell you like his Chinese is better than this one, this one is better than that one in terms of skills practice* *,* *so we feel bad. (P1).*

International undergraduates were often paired with Chinese nursing students during clinical practice. The diligent learning attitude of some Chinese interns often caused anxiety and unease among the participants.

*Look at someone who is more hard-working than you. That’s just the only disadvantage about being paired with a Chinese student. (P11)*

### Accepting challenges

“Accepting challenges” describes the participants’ transition to confronting and embracing difficulties. Upon realizing that obstacles were inevitable, the African nursing students felt they had no choice but to accept them. Initially, the students still felt a sense of resignation, but through self-adjustment, their tolerance for challenges increased. Over time, the difficulties became less overwhelming, allowing the students to actively accept them. Furthermore, by recognizing the benefits of these difficulties, the participants could embrace them with inner peace.

#### Having to accept

As the internship progressed, participants gradually acknowledged that clinical practice was not always smooth and that challenges were inevitable. They reluctantly accepted the reality that most patients and their families communicated in Chinese and that not all patients were friendly.

*They (the patients and* their family members*) all speak Chinese. So it’s about the realization of being in reality and knowing where you are and what you’re supposed to do. (P9).*

Despite accepting their situation, the students still felt distressed. They sought ways to regulate their emotions and resolve their internal conflicts. Some participants told themselves that complaining was merely an excuse for not wanting to learn and that success could only be achieved through persistent effort. Others reminded themselves of their initial goals in studying abroad, emphasizing that these challenges should not hinder their academic pursuits.

*We are definitely going to come across challenges one way or another. But we should always know their aim and stay focused on what we want to achieve originally. (P11)*

#### Being able to accept

Over time, as participants’ proficiency in Chinese improved and their relationships with patients became more positive, they found that the challenges, such as language barriers and embarrassed identities, became less difficult to accept.

*At the end of the day, I accept Chinese. So, it becomes much easier for me. Language is not such a barrier. (P2)*

When the African students realized that these challenges were opportunities to become the better versions of themselves, they began to accept these challenges actively. In addition, when some participants realized that patients needed their help and care, they were motivated to embrace and persist through the challenges.

*They were my motivation. My patients which are my motivation every day. (P13)*

### Making personal efforts

“Making personal efforts” refers to the proactive steps participants took to address and overcome the challenges they encountered. They actively managed their self-image and tried to present a positive impression to both patients and preceptors to gain their trust and support. The participants also engaged in thoughtful self-reflection and practiced diligently to improve themselves and adapt to the difficulties.

#### Self-image managing

The African nursing students presented a friendly attitude toward the Chinese patients by proactively greeting them and expressing care. They believed that, upon experiencing their friendliness and sincerity, patients would be more open and supportive.

*So maybe every morning you can say “good morning” or “how are you doing”. By consistently doing this for a week, they will start becoming close to you. (P9)*

Some participants also exhibited a studious image during their internships by being punctuality, showing eagerness to learn, and having the courage to ask questions. This proactive and positive attitude was viewed as a way to earn appreciation from their preceptors, leading to more learning opportunities and guidance.

*You are always willing to learn, willing to working, willing to do whatever they are asking you to do without complaining. Then you will have more opportunity with this teacher. And then this teacher will communicate with you more. (P7)*

#### Self-reflecting and improving

The participants engaged in regular self-reflection after each day of clinical practice. They believed that it was crucial to reflect on their clinical experiences and avoid repeating past mistakes.

*Failing is normal. You fail and stand up again. So it’s just a matter of learning from the same mistake. (P1)*

After identifying areas for improvement, the students actively engaged in targeted practices, such as repetitive simulations of nursing procedures and honing their Chinese language skills, to continuously improve themselves. Some participants reported that consistent practice increased their confidence.

*I think practice makes perfect,,, the more practice we engage in, the greater our confidence becomes while doing it. (P7)*

### Being not alone

“Being not alone” highlights the support and companionship participants experienced during their clinical practice. They received assistance from various sources, including clinical preceptors, fellow students, and patients.

#### Preceptors are strong backings

The participants reported that preceptors patiently explained concepts, addressed doubts, and corrected mistakes. Regular meetings were also arranged to solicit and incorporate student feedback into nursing education management.

*I always have a lot of questions, and then I’m glad that teachers are not annoyed. (P6)*

The warmth of the preceptors fostered a sense of belonging among the participants. Preceptors also provided solace during emotionally challenging times, such as when students struggled with unsuccessful nursing procedures or dealt with patient refusals.

*No, I can’t do it, but she never gave up on me, encouraged me I can do it. (P3)*

#### Mutual assistance among fellow students

The participants frequently engaged in discussions with their peers, sharing challenges and mutually benefiting from each other’s learning experiences. Their similar cultural backgrounds and educational trajectories allowed them to empathize with each other.

*She also helped me where I didn’t understand. So we used to help each other. (P1)*

The participants also formed close bonds with Chinese nursing students during their clinical internships, with several highlighting the Chinese interns’ helpfulness and willingness to assist. Furthermore, some reported mutual benefits, particularly for the Chinese interns in improving their English proficiency.

*Maybe like one pair and one pair… She teaches me Chinese, I teach her English, which is beneficial to everyone. (P6)*

### Patients provide trust and warmth

As the students proactively engaged with patients, harmonious patient-nurse relationships developed. Patients began to request that the students perform nursing procedures on them and provided encouragement and additional opportunities for the students when nursing procedures were unsuccessful.

*The patients gave me the other hand, and told me to try in this hand. They also told me “you can do it, you can do it”. (P13)*

Many participants mentioned forming close friendships with patients. Patients’ warm behaviors, such as presenting handmade gifts and making special visits to them, all deeply touched the participants.

*He drew my country’s flag for me as a gift. I was very touched. I was very happy. He gave it to me because I have been giving him medication every day. (P4)*

### Growing up as a real nurse

“Growing up as a real nurse” reflects the participants’ experience of transitioning from students with theoretical knowledge to becoming capable nurses. Throughout the clinical internship, the participants accumulated extensive clinical knowledge, developed proficient nursing skills, and gained a high level of professional nursing qualities.

#### Increasing nursing knowledge and skills

During their clinical internships in China, the participants were exposed to a wealth of advanced nursing knowledge, modern medical equipment, and traditional Chinese medicine practices, such as acupuncture and cupping therapy, which were often unavailable in their home countries.

*I learned a lot about traditional Chinese medicine such as acupuncture, cupping, Tuina. It’s like magic, yeah, really magic. (P3)*

By the end of their clinical internships, the nursing students, initially plagued by frequent failures and anxiety, became capable of handling complex tasks, with some even performing certain procedures independently. This progress significantly contributed to the participants’ sense of achievement.

*I was happy, I was like I did a great achievement cause I thought it’s hard, I thought I could not do it, but I finally did it. (P12)*

#### Enhancing nursing professional quality

The clinical internships instilled in the participants the importance of patience. They learned to communicate patiently with patients and their families, even during busy clinical periods. Additionally, the participants developed greater compassion and empathy, which enabled them to better understand patients’ needs.

*I wasn’t someone who could empathize with people. But now I have grown that kind of empathy and compassionate through this internship. (P6)*

Due to the challenges encountered, several participants reported that they had become mentally stronger and more resilient in their approach to future clinical work. In addition, by working with other healthcare staff, the participants developed a greater appreciation for the significance of teamwork and further enhanced their collaborative skills.

*It has also taught me teamwork, what we are to be able to work with other people… built my teamwork skills. (P11)*

## Discussion

To the best of our knowledge, this is the first qualitative study exploring the clinical internship experiences of African undergraduate nursing students in China. These students encounter numerous challenges during their internships due to the language barriers, culture differences and other contributing factors. This is similar with the experiences of other international nursing students who undertake clinical placements in Western countries [13, 17]. However, our study further reveals the process through which the African students achieve positive changes after overcoming these challenges, which is rarely reported in previous studies. Accepting challenges, making personal efforts, and receiving support from others are crucial aspects of this process and play a pivotal role in the students’ positive development.

The primary challenge during clinical internships was the language barrier, which hindered participants’ ability to effectively engage in nursing activities and induced a sense of alienation [6, 13]. Our study further found that language-related issues were especially prominent in the early stages of internship. This indicated that the students might lack sufficient language preparation before clinical practice, underscoring the importance of early exposure to the clinical language and the need to strengthen ward-based communication skills training programs [18, 19]. For example, more nursing courses taught in Chinese could be incorporated, with a particular focus on idioms, phonetics, and accent reduction [20]. Furthermore, staring pre-internship training earlier and offering these experiences more frequently could help the students become aware of potential challenges they may face in advance.

This study found that the identity of “international nursing students” posed some challenges for the participants, as they were subject to attention, comments, suspicion and rejection from patients, leading to feelings of discomfort and inferiority. This is consistent with previous studies [9, 21]. However, students were not always inclined to discuss their negative experiences with clinical instructors [13]. Thus, the preceptors should regularly assess students’ psychological well-being. Furthermore, with the globalization of medical education, an increasing number of international nursing students will come to China for clinical practice. It is crucial to develop programs to help patients embrace diversity and reduce their stereotyping of the international nursing students [10]. In addition, the maladjustment and negative emotions of African nursing students may be related to their relatively low intercultural interpersonal competency, which is a basic skill and an important element of international talent training [22]. However, there has been limited research in this area, indicating a need for further investigation.

Although participating in clinical internships alongside other excellent international and domestic nursing students offers certain advantages, such as motivating individuals to work diligently and providing opportunities to practice language [17, 23, 24], it also poses challenges for some African students. Specifically, they may experience peer pressure. According to social comparison theory, engaging in upward comparisons with more competent individuals can negatively affect on one’s self-esteem [25]. This is particularly evident when preceptors publicly praise the outstanding performance of certain students, which can induce feelings of inadequacy among those who are not recognized. This phenomenon may also be related to cultural differences. In Chinese culture, praise is characteristically public and explicit, ensuring that both the recipient and others are fully aware of it [26]. However, the praise bestowed by preceptors on others made African students feel that their own efforts and capabilities were overlooked. Thus, it is important for Chinese preceptors to reconsider their way of expressing praise and ensure that all students feel valued during their clinical internships.

Accepting challenges has a positive impact on study abroad adaptation [27]. Consistent with previous study [7], our findings indicate that a recognition of the importance of internship and a strong sense of professional identity are positive inner motivators that help African nursing students accept challenges and adapt to their internships. However, our study further found that reminding students of their goals for studying abroad is also an effective strategy. This likely motivates students to strive for desirable learning outcomes [28]. In particular, the students in our study come from developing countries, and their families may bear significant financial burdens to support their study abroad. These goals can be seen as responsibilities that push them to study diligently and accept challenges.

Our study found that the participants received substantial support during their clinical internships. This might be related to China’s emphasis on collectivism [14] and the traditional Chinese cultural value of “reqing haoke” (i.e. welcomingness and hospitableness) [29]. Interestingly, we further revealed that the students’ positive attitude and behaviors might contribute to the support they received. According to social exchange theory, interpersonal interactions are reciprocal [30]. The students’ proactive pursuit of knowledge, continuous self-reflection, and friendly disposition led to positive responses from their preceptors and patients. A harmonious working atmosphere facilitated the adaptation of African nursing students to clinical practice [7], thereby creating a positive feedback loop. Further research is necessary to explore the bidirectional interaction we observed between African nursing students in China and their preceptors or patients.

This study has some limitations. First, the participants were all recruited from one city in southeast China. Considering that different regions might impact the clinical internship experiences of international undergraduate nursing students, the results of this study should be further explored with students from other regions. Second, most of the participants were female and had a clinical internship experience of over 5 months, which somewhat limited the diversity in this study.

## Conclusion

Faced with various challenges during their clinical internship, the African students actively adjusted themselves and embraced difficulties. They employed a range of strategies to address these challenges, relying on their own resilience and receiving help from others, ultimately achieving positive changes. Future research could adopt a longitudinal design to explore the issues faced by students at different stages of their internships. Additionally, future studies could also explore the perspectives of preceptors and patients to enhance the clinical practice satisfaction of African nursing students from different angles.

## Data Availability

The data are not publicly available due to privacy or ethical restrictions. The data that support the findings of this study are available on request from the corresponding author on reasonable request.
